# Replicated biopolymer pattern on PLLA-Ag basis with an excellent antibacterial response

**DOI:** 10.1016/j.heliyon.2023.e21566

**Published:** 2023-11-02

**Authors:** Bára Frýdlová, Dominik Fajstavr, Nikola Slepičková Kasálková, Silvie Rimpelová, Vladimíra Svobodová Pavlíčková, Václav Švorčík, Petr Slepička

**Affiliations:** a Department of Solid State Engineering, The University of Chemistry and Technology Prague, 166 28, Prague, Czech Republic; b Department of Biochemistry and Microbiology, The University of Chemistry and Technology Prague, 166 28, Prague, Czech Republic

**Keywords:** Organic coating, Nanopattern, Nanostructures, Silver nanoclusters, Antibacterial properties, Replication, Hot-embossing, poly(l-lactic acid), PDMS, Soft-lithography, Nano-lithography

## Abstract

The design of functional micro or nanostructured surfaces is undergoing extensive research for their intriguing multifunctional properties and for large variety of potential applications in biomedical field (tissue engineering or cell adhesion), electronics, optics or microfluidics. Such nanosized topographies can be easily fabricated by various lithography techniques and can be also further reinforced by synergic effect by combining aforementioned structures along materials with already outstanding antibacterial properties. In this work we fabricated novel micro/nanostructured substrates using soft lithography replication method and subsequent thermal nanoimprint lithography method, creating nanostructured films based on poly (l-lactic acid) (PLLA) fortified by thin silver films deposited by PVD. Main nanoscale patterns were fabricated by replicating surface patterns of optical discs (CDs and DVDs), which proved to be easy, fast and inexpensive method for creating relatively large area patterned surfaces. Their antimicrobial activity was examined *in vitro* against the bacteria *Escherichia coli* and *Staphylococcus epidermidis* strains. The results demonstrated that nanopatterned films actually improved the conditions for bacterial growth compared to pristine PLLA films, the novelty is based on formation of Ag nanoparticles on the surface/and in bulk, while silver nanoparticle enhanced and nanopatterned films exhibited excellent antibacterial activity against both bacterial strains, with circa 80 % efficacy in 4 h and complete bactericidal effect in span of 24 h.

## Highlights

• Micro/nanostructured substrates using soft lithography replication method were prepared.
• Imprinted nanostructured PLLA was enhanced with silver.
• Thermal stress during replication led to formation of isolated Ag nanoclusters in PLLA.
• Strong antibacterial properties of replicated PLLA-Ag composite was confirmed.
• Possibility of large scale manufacturing of PLLA-Ag nanogrooved replicas.

## Graphical abstract

**Figure:**
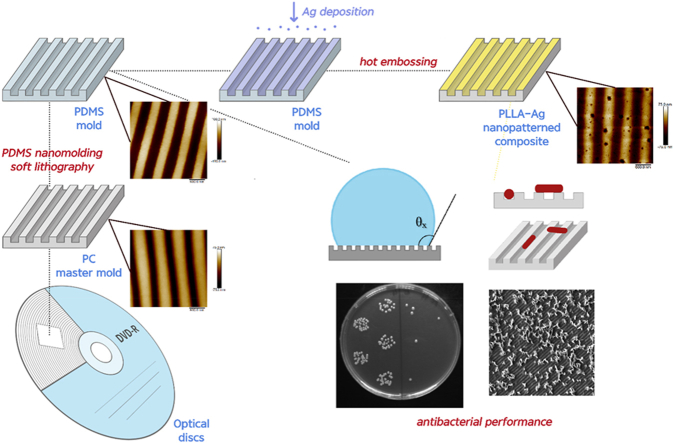

## 1 Introduction

Nano-structures on natural surfaces fascinated researches thanks to their unique properties for decades. Thanks to the development of scanning electron microscope (SEM) in the mid-1960s, the secret of self-cleaning and superhydrophobic mechanism of lotus leaf was finally revealed [1]. From that point on it was possible to explore micro and nano-structured topography of many natural surfaces and the potential of future fabrication of novel structures and their implementation in everyday use. The fabrication of various micro and nano-patterned materials started by examining and mimicking natural structures such as butterfly's [2] or cicada's [3,4] wings, lotus [5] and rice [6,7] leaves, gecko's feet [8] or shark skin [[9], [10], [11], [12]], etc. [13]. Through millions of years of evolution, many of these surfaces developed fairly periodic micro-to-nanoscale hierarchical features as protection against environmental conditions. For example, some surfaces decrease adherence and lower proliferation rate of bacteria, spores or contaminating particles and therefore can be characterized as either antibiofouling or antibacterial [14]. Moreover, surfaces like cicada's wings are able to mechanically rupture and kill attached bacteria by the action of their specific surface nanoarchitecture, therefore are not actually antibiofouling but bactericidal [4]. Such structures can serve as an inspiration for artificial reproduction (with various results) or simple replication using naturally occurring surfaces as molds. Large number of bioengineering studies have aimed to reproduce mainly the antibacterial behavior of certain natural surface structures using wide variety of micro or nano-fabrication methods. By developing range of chemical and mechanical methods, such as various types of lithography: photolithography, electron beam lithography (EBL), proton beam lithography (PBL), ion beam lithography (IBL), ultraviolet (UV) lithography, X-ray lithography, nano-imprint lithography (NIL), colloidal lithography, capillary force lithography (CFL) and several others [13,15], fabrication of wide variety of hieararchely nano-structured surfaces can be achieved. Lithography techniques are generally based on copying surface pattern from a master mold and transferring it to another surface.

Soft lithography is group of patterning techniques utilizing soft/elastomeric stamps or molds used for fabrication or replication of surface micro- or nanostructures for many applications especially in bioengineering. This method combines molding, imprinting and embossing with elastomeric stamps. Soft lithography is also relatively procedurally simple, low-cost, high throughput and the pattern resolution ranges from nanometer to micrometer precision. The only drawback is the dependence of soft-lithography on other advanced techniques for master mold fabrication. However, once the master mold is obtained, it can be repeatedly used for production of the elastomeric molds. The most commonly used material for mold fabrication is poly (dimethylsiloxane) (PDMS), a biocompatible, transparent, flexible, gas permeable and naturally hydrophobic elastomer. In this fabrication method, the viscous PDMS precursor is usually poured over master mold, degassed in vacuum, heated to cross-link (cure) and subsequently peeled off of the master mold.

Nano-imprint lithography technique can be divided into two broad classes: thermal NIL and ultraviolet (UV)-NIL, both of which are used frequently. Thermal nano-imprint lithography, also known as hot embossing, is one of the most cost effective and high volume replication technologies to precisely transfer microstructure patterns from a master mold to a thermoplastic polymer substrate at given pressure and above polymer's glass transition temperature (*T*_*g*_). It is an important nanolithography method used to replicate nanofeatures and nanopatterns through a thermal nanoimprint process and provides a high-throughput route for the fabrication of structured substrates used as contact cell growth guidance in tissue engineering. Hot embossing is suitable for patterning wide range of thermoplastic polymers and is especially convenient for polymers degradable in solvents, water, etc. Since it's dry process employing only elevated temperature and pressure. Typical hot embossing process consists of four major steps: (i) heating the mold and substrate to working temperature, (ii) embossing desired patterns at embossing temperature, (iii) cooling the mold and substrate to demoulding temperature, (iv) demoulding the patterned substrate. Generally, the most important parameters for hot embossing are temperature (mostly glass transition temperature *T*_*g*_), forming pressure and holding time [16]. Disadvantages of NIL are the pattern size limitations and cost and fabrication of master molds, though if not damaged, the mold can be repeatedly reused, thereby further reducing the over-all expenses.

Several bio-mimicking high aspect ratio surface structures were developed by NIL techniques and can be found in the literature: nanocones [17,18], nanopillars [19,20], nanospikes [21], nanoneedles [[22], [23], [24]], micro lines, micro mushrooms [25,26], groove arrays [27] etc. Combining various lithography methodologies can also lead to fabrication of hierarchical multi-scale features [28]. Recently, more research attention has been paid to development of novel hierarchical and nanostructured material surfaces. These surfaces with unique morphologies can exhibit excellent and advanced properties, such as superhydrophobicity [[29], [30], [31]], anisotropic wettability [32], antireflectivity [[33], [34], [35]], adhesion [8] or antibiofouling [12] and therefore can be suitable for application in microelectronics, optics or optoelectronics, biosensors and especially in medicine and tissue engineering, e.g. antibacterial properties of polygon or nano-pillar patterned orthopaedic implants [13,36]. Focus of surface modification is especially prevalent in biomedical applications [22], where periprosthetic bacterial film formation may lead to implant rejection or may cause extreme immune reaction or infection, which then must be overcome with antibiotics [13,37]. Nanotexturing of implants can be great solution to prevent viable pathogenic bacterial contamination and attachment, effective way to avert multi-species microorganism biofilm development and also to avoid excessive use of antibiotics. However, such surfaces often need to maintain affinity to cells to ensure desired (host) tissue attachment and therefore must provide selective bioactivity and non-toxicity. Already conventionally established approach in biomedicine is introduction of biocidal agent coatings, such as antibiotics, drugs or silver [18], therefore combination with nanotexturing may produce a synergistic action and enhanced antimicrobial performance.

Poly (l-lactic acid) (PLLA), is biobased and widely researched aliphatic thermoplastic polyester, mass produced as dl-lactide monomer by the microbial fermentation of agricultural by-products. PLLA is common biopolymer used in medicine and especially in tissue scaffolds or implant engineering, due to its great characteristics, such as biocompatibility and biodegradation, transparency, great mechanical properties, stability, etc. [38]. These properties make PLLA a great candidate for use in many industrial fields, e.g. in the pharmaceutical industry, biomedicine, textile industry and for current use for food packaging. Extensive research is focused especially on enhancing already great properties of neat PLLA and overcoming some of its shortcomings, such as low thermal stability, toughness or gas barrier properties and tailoring them for specific use [39]. PLLA nanocomposites are in the forefront of interest, not only for improving but also introducing new characteristics, such as antibacterial or antioxidant properties, when combined with ideal or active agents. Silver nanoparticles are known for distinct bactericidal properties and are well established in medicinal use. Silver-based coatings has been used to numerous medical applications, such as catheters, drains or wound dressings [40,41]. Short-term and low concentration biocompatibility has been observed, though high concentrations of silver have demonstrated damaging effects on tissues *in vitro* studies and the long-run safety remains to be determined. The antibacterial properties of Ag are affiliated to the nanoparticle's slow oxidation and subsequent release of highly potent Ag^+^ ions from metallic silver in moist environment [40]. The natural affinity to sulphur proteins and electrostatic attraction may lead to silver ion adherence to the cell wall and cytoplasmatic membrane, causing increase in cytoplasmic membrane permeability which can induce bacterial envelope disruption. Uptaken free silver ions can denaturate ribosomes in the cytoplasm thus inhibiting protein synthesis, or deactivate respiratory enzymes generating reactive oxygen species, that can serve as a prime agent of membrane disruption and DNA modification. Interaction of silver with sulphur and phosporus in DNA can also create issues in DNA replication, cell reproduction and can also lead to microorganism demise. Nanosilver is effective against most viruses and bacterial strains (both Gram-positive and Gram-negative) and due to its mechanism of action microorganisms are not likely to gain resistance in the mutation process, therefore use of silver can be future solution of ongoing antibiotic resistance crisis. By decreasing the size of nanoparticles the viable surface area increases (compared to bulk material) and greater membrane permeability is provided, since not only silver ions, but also silver nanoparticles themselves possess the ability to penetrate the cell wall. Such nanoparticles can accumulate in the cell and subsequently lead to denaturation of cell or cytoplasmatic membrane, repturing organelles and resulting in cell lysis. Antibacterial effect is closely associated with size, shape and charge of nanoparticles, manifesting in different approach to bacterial wall adhesion and subsequent bacterial membrane damage and intracellular organelle disruption. Nanoparticles smaller than 10 nm have larger specific surface area, better membrane permeability and faster Ag^+^ ion dissolution thus exhibit significantly better biological activity [41].

Especially the development of antimicrobial surfaces has risen to the forefront of interest, as a possible solution for ongoing antibiotic-resistance crisis. Many recent studies were focused on development of alternative approach to prevent bacterial attachment by creating micro and nano-textured surface structures, inspired by naturally occurring antibacterial and antibiofouling surfaces, as a promising way of passive bacterial infection control in biomedical field. Combination of nanostructured surfaces and silver is promising surface alteration approach resulting in synergic action and enhanced antibacterial performance. The novelty of the presented study consists of development of simple fabrication method of nanostructured PLLA surfaces and mainly of introduction PLLA-Ag composite with enhanced surface properties. In addition, this paper provides comparison of antibacterial properties between non-structured, structured and Ag-deposited (enhanced) surfaces.

## 2 Experimental

### 2.1 Materials

Polydimethylsiloxane (PDMS) elastomer kit Sylgard 184® with the base/curing agent ratio 10:1 was purchased from Sigma Aldrich (Merck, USA). Commercially available compact disc-recordable (CD-R) (Verbatim, 700 MB) and digital versatile disc-recordable (DVD-R) (EMTEC, 4.7 GB) were purchased from the market. The silver targets for diode sputtering were purchased in 99.99 % purity from Safina s. r.o. (Czech Republic). Sheets of poly (L-)lactic acid (PLLA) biopolymer films in 0.05 mm thickness were purchased from GoodFellow (UK).

### 2.2 Preparation of optical discs master molds

The patterned polycarbonate (PC) parts of two commercially available optical discs CD-R and DVD + R were used as initial masters. Small circles (50 mm in diameter) were cut out of CD-R and DVD-R respectively and the PC layers were separated. For the CD-R disc the reflective and printing layers were peeled off using scotch tape. The organic dye layer was dissolved by 30 min ultrasonic treatment in ethanol, and after repeated rinsing with distilled water and ethanol a grooved PC layer was obtained. The DVD-R disc consists of two PC surfaces with reflective and dye layer in between them. The two PC layers can be manually separated along the cut edge by tweezers. The grooved PC layer was than immersed in methanol and sonicated for 30 min to remove excess reflective layer, dyes and any dust particles. After repeated rinsing with distilled water and methanol the DVD-R master mold was obtained.

### 2.3 Fabrication of PDMS molds

**Fig. 1:**
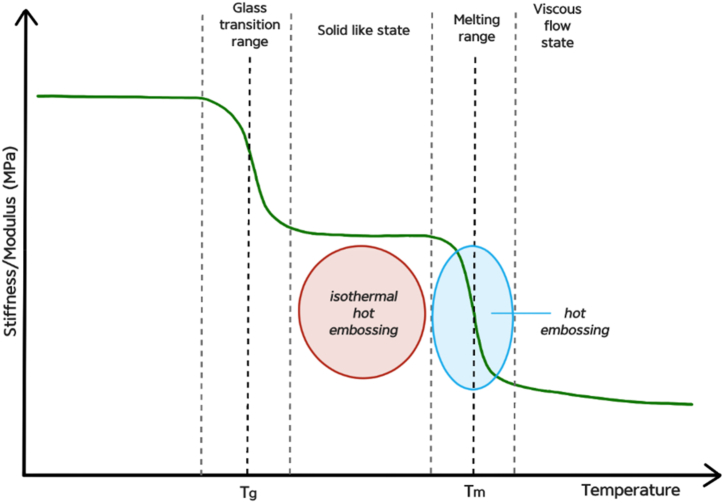
Illustrative graph of modulus versus temperature for a semicrystalline polymer with molding windows for hot embossing.

PDMS was used to replicate the surface structure of CD and DVD discs by soft lithography (cast-molding) technique (see Fig. 1). The mixture of PDMS base and curing agent was thoroughly manually mixed in weight ratio of 10:1 and then poured over PC master molds placed in Petri dishes. After subsequent degassing in vacuum chamber for 1 h, the Petri dishes were thermally annealed in an oven heated at 80 °C for 2 h for completing cross-linking of the polymer. Once the thermoset was cured the flexible negative mold was carefully peeled off of the PC master.

### 2.4 Thin film deposition

The thin metal films were deposited onto PDMS molds by physical vapour deposition (PVD). Deposition was performed by diode sputtering from 99.99 % Ag target (supplied by Safina, Vestec, Czech Republic) using Bal-Tec SDC 050 device (BalTec Maschinenbau AG, Pfäffikon, Switzerland). The sputtering conditions were a total pressure about 5 Pa of Ar (99.995 % purity), current of 15 mA and distance of 60 mm between electrodes. The deposition was performed at room temperature, and the deposition times varied between 100 and 300 s to create layers of different thicknesses.

### 2.5 Hot embossing

The pre-formed PLLA film was placed on the PDMS metal deposited mold and thermally annealed in a Binder oven with a thermostat at 180 °C for 15 min without employing any pressure. Teflon foil was added as underlying layer, since its able to withstand high temperatures and is preventing sticking of the melted polymer to the glass surface of Petri dishes.

**Fig. 2:**
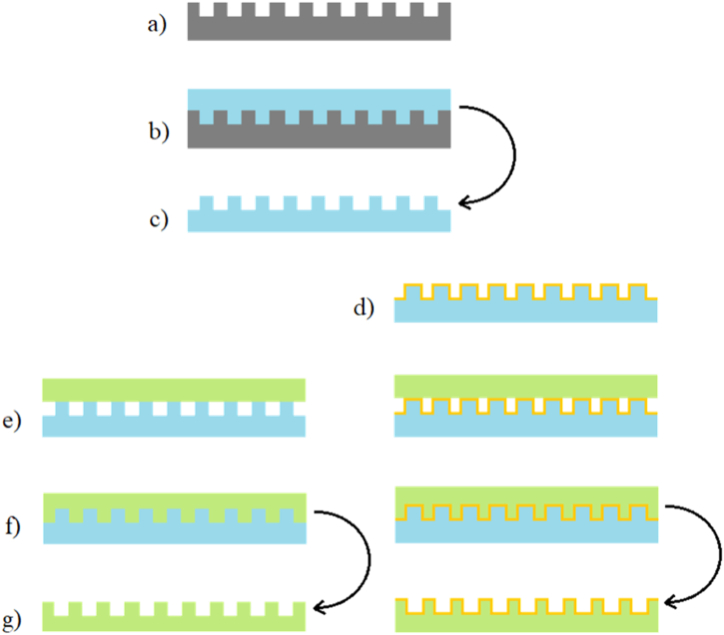
Schematic illustration of fabrication of PLLA based nanopatterned surfaces. (a) PC optical disc – master mold, (b) PDMS soft lithography, (c) elastic PDMS mold, (d) deposition of silver layer on PDMS mold, (e) PLLA placement on PDMS mold, (f) hot embossing, (g) PLLA/PLLA-Ag nanopatterned film.

After cooling in the air at the room temperature (25 °C) the PLLA film was manually peeled off of the flexible mold. Although the glass transition temperature T_g_ for PLLA is ∼50–80 °C the temperature used for this method was chosen to be even above melting temperature T_m_ (∼170–180 °C) to get the polymer to viscous flow state (schematically see Fig. 2). Temperatures are mentioned as intervals since the degree of crystallinity of this semi-crystalline thermoplastic determines its thermal properties.

### 2.6 Analytical methods

Surface morphology and roughness of the replicated, embossed and Ag enhanced films were examined by atomic force microscopy (AFM) technique using Dimension ICON (Bruker Corp., Billerica, MA, USA). The samples were analyzed in Scan-Assyst® mode using nitride lever SCANASYST-AIR with Si tip (spring constant of 0.4 N m^−1^). NanoScope Analysis software was applied for data processing. Surface roughness (*R*_*a*_) represents the arithmetic mean of the absolute values of the height deviations measured from the central plane.

The morphology of the samples surfaces was also characterized by complementary technique using the scanning electron microscope FIB-SEM LYRA3 GMU (Tescan. Brno, Czech Republic). The acceleration voltage was set to 10 kV. To ensure the conductivity of the samples, their metallization was performed using sputtering technique (Quorum Q300T) by deposition of Pt layer (thickness of 20 nm, Pt target, purity of 99.9995 %). The elemental composition was measured by energy-dispersive X-ray spectroscopy (EDS, analyzer X-ManN, 20 mm^2^ SDD detector, Oxford Instruments, United Kingdom), while the accelerating voltage for SEM-EDS analysis was set to 10 kV.

The elemental composition on the material surface was analyzed by X-ray photoelectron spectroscopy (XPS) using spectrometer ESCAProbeP (Omicron Nanotechnology Ldt., Taunusstein, Germany). As a source, a monochromatic X-ray at an energy of 1486.7 eV was used. Atomic concentrations of elements were determined from the individual peak areas using CasaXPS software.

Wettability of the studied samples was determined by measurement of contact angles (CA, θ) on goniometer Advex Instruments (Brno, Czech Republic) connected to the SEE System 7.1 program. Analysis of CA was performed at room temperature with 8 μL drops of distilled water (dyed with methyl violet) using a Transferpette® automatic pipette (Brand, Wertheim, Germany) at 6 different positions of 3 samples in parallel and perpendicular direction. Subsequently, the drops were photographed and evaluated by 3 marked points.

Absorption spectra were measured using PerkinElmer instrument, Lambda 850+ spectrometer type. The spectrometer has wavelength range of 190–1100 nm and is therefore suitable for both solutions and solid samples.

### 2.7 Antibacterial study

Antibacterial activity of nanostructured and silver enhanced PLLA foils was determined by drop method using gram-negative and gram-positive bacterial strains of *Escherichia coli* and *Staphylococcus epidermidi*s, respectively. The bacterial strains were inoculated from agar plates into liquid Luria-Betrani (LB) medium and subsequently cultivated for 16 h at 37 °C in an orbital shaker. Then, the optical densities of both bacterial cultures were determined at 600 nm, after which they were serially diluted. The amounts of 2∙10^−4^ and 4∙10^−4^ of colony forming units (CFU) of *E. coli* and *S. epidermidis*, respectively, were inoculated per 1 mL of sterile phosphate-buffered saline (PBS, pH of 7.4), into which were the evaluated samples immersed. The size uniform samples were then gently mixed and dynamically incubated at 24 °C for 1, 4 and 24 h. Next, the samples were mixed again and 20 μL drops of each sample (5 biological replicates) for both bacterial strains were pipetted onto LB agar plates 3 times. The plates were than cured at 37 °C for 24 h, after which the CFU number was counted and compared with the number of CFUs on control plates (bacteria on agar plates incubated for the same time period only in PBS without any evaluated sample). The experiments were performed in sterile conditions.

## 3 Results and discussion

### 3.1 Surface morphology of the PDMS-optical disc replicas

**Fig. 3:**
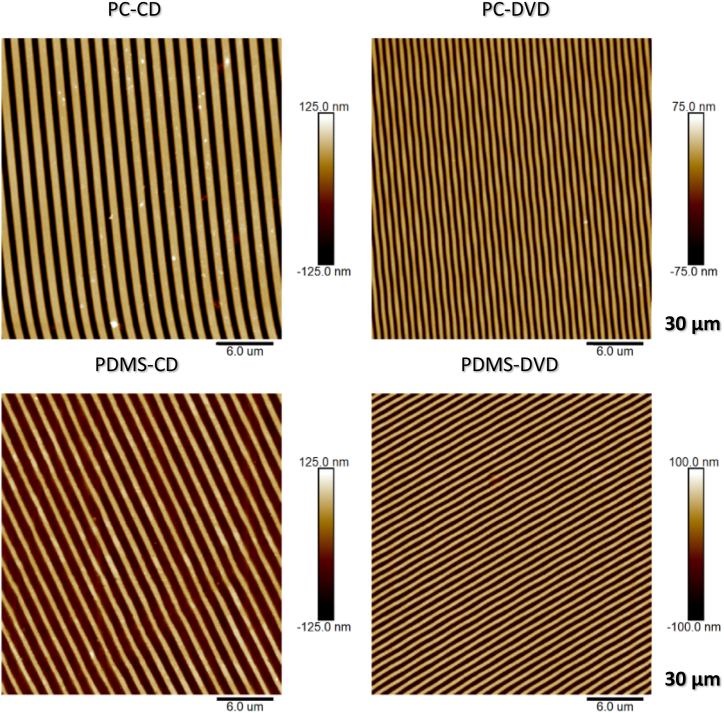
Surface morphology of polycarbonate optical discs (CD-R, DVD-R) (top row) and their PDMS replicas (bottom row). Squares 30 × 30 μm determined by atomic force microscopy.

The first step in this work was to characterize the surface morphology of the polycarbonate master molds and subsequently fabricated flexible PDMS molds, evaluate precision of the replication and the dimensions of formed periodic pattern. Nanometer scale groove arrays on the PDMS polymer were fabricated by soft-lithography technique replicating structures from optical discs, as shown on AFM images by Fig. 3 (AFM images of original discs and PDMS replicas). The structures in top row present the morphology of the original PC master molds (cleaned optical disc) and the images in the bottom row show flexible PDMS molds obtained by their replication employing soft lithography method in experimental part. Their typical parameters and surface changes are summarized in Table 1 bellow.

**Table 1:** Parameters of replicated CD and DVD patterns and their masters.

Sample	Groove height h (nm)	Center to center distance d (μm)	RMS roughness (nm)	Surface area S (increment) (%) (3 μm)
CD – PC master	173.6 ± 1.8	1.5 ± 0.03	74.1	9.0
CD – PDMS replica	133.1 ± 6.7	1.5 ± 0.04	53.2	7.9
DVD – PC master	101.8 ± 2.4	0.7 ± 0.02	37.0	6.8
DVD – PDMS replica	128.4 ± 2.7	0.7 ± 0.02	50.8	12.9

It can be concluded, that nanogroove pattern was fabricated on PDMS over large area with high uniformity and high aspect ratio. The typical CD-R and DVD-R based nanogrooves on PC had approximately 170 and 100 nm depth and 1.51 and 0.74 μm periodicity, respectively. The negative PDMS replicas exhibited high fidelity in dimensional replication. Both replicas displayed nanogrooves with depth of approximately 130 nm and virtually the same period as the PC masters, although with reversed dimensions of grooves and ridges. In case of CD-R PC master molds, the ridge and groove width were 900 and 460 nm, respectively, whereas for the PDMS replicas, ridge width was approximately 445 nm and groove with was 850 nm. Dimensions of the DVD-R PC master molds were approximately 450 nm and 300 nm in ridge and groove width, respectively and the PDMS replica exhibited 320 nm and 420 nm ridge and groove width. Surface structure, dimensions and periodicity also play pivotal role in surface wettability, along with surface chemistry and energy, as will be described further.

### 3.2 Surface morphology of the PLLA replicas (morphology and wettability of structured surfaces)

We have chosen a relatively cheap, commercially available and biocompatible PLLA, because of its already extensive use in medicine and tissue engineering. PLLA substrates can be structured without modifying the material composition and also without applying additional surface chemical treatments. One of PLLA's noteworthy characteristics is relatively low thermal expansion while melting, which is main reason for its use as 3D printing material, therefore seems to be perfect candidate for precise replication employing high temperatures.

**Fig. 4:**
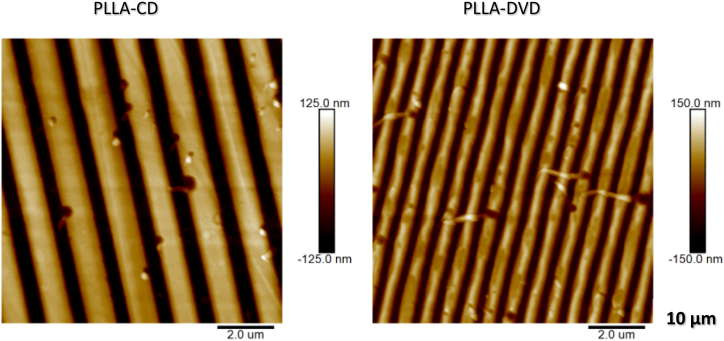
Surface morphology of nanostructured PLLA films, replicating CD-R and DVD-R surface patterns from PDMS mold via hot embossing method, determined by atomic force microscopy. (2D 10 × 10 μm squares).

During nanostructuring, periodic patterns replicated by PDMS molds were subsequently transferred onto PLLA films via hot embossing method. The resulting films exhibited relatively high aspect ratio and uniformity over a large area, disrupted only with occasional presence of trapped air bubbles or dust particles. Fig. 4 shows, the AFM images of PLLA replicas of both optical discs.

Dimensions of the patterns transferred to PLLA were approximately 165 nm in ridge height for both disc replicas and periodicity of 1.49 and 0.75 μm for CD and DVD, respectively. For the CD-R PLLA based replica, the typical width of the grooves and ridges were 385 and 900 nm, respectively. The DVD-R based PLLA replica exhibited 160 nm wide grooves and 455 nm wide ridges.

**Fig. 5:**
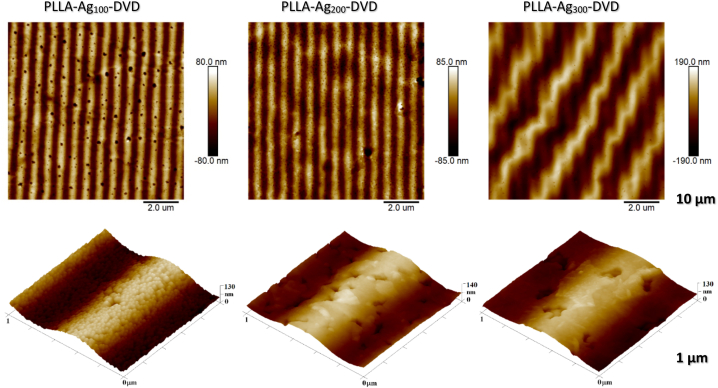
AFM images of surface morphology of silver enhanced PLLA replicas with DVD pattern. While upper row presents 10 × 10 μm scans, the bottom row shows surface details on 1 × 1 μm images, for samples with different silver sputtering times.

To create silver enhanced PLLA films with surface patterns, the thin silver films was deposited on PDMS mold via diode sputtering. By the nature of PVD process the thin layer is established “atom-by-atom”, which makes it almost perfect replica of the master geometry. However, this method is limited mostly by the maximum obtainable thickness of the deposited films, by deposition velocity, but also by the substrate geometry. PDMS does not, however, provide great adherence for the deposited films, which can be removed simply by scratching or using scotch tape. By carrying out the hot embossing of PLLA, the metal film can be, on the other hand, easily detached and incorporated onto the melting surface of PLLA film. During annealing, the metal films also undergo changes and in particular conditions can form globular silver clusters, as is evident in Fig. 5. The brief profligacy of the non-continuous metal film on the elastic and antifouling PDMS surface before attachment to the PLLA surface seems to be crucial step for nanoparticle formation. By simple deposition of the metal films of the same thickness directly on the PLLA film and subsequent annealing in the same conditions, the nanoparticles do not form.

The resulting parameters and surface changes of nanostructured PLLA samples enhanced with various silver layer thicknesses are summarized in Table 2 bellow.

**Table 2:** The summary of feature size and surface changes of nanostructured PLLA surfaces. Their feature size was analyzed according to AFM images.

Sample	Groove height h (nm)	Center to center distance d (μm)	RMS roughness (nm)	Surface area S (increment) (%) (3 μm)
**PLLA – DVD**	165.2 ± 16.6	0.8 ± 0.08	60.0	15.9
**PLLA-Ag**_**100**_**-DVD**	80.7 ± 2.0	0.8 ± 0.02	28.5	16.6
**PLLA-Ag**_**200**_**-DVD**	78.5 ± 3.4	0.8 ± 0.02	27.2	11.1
**PLLA-Ag**_**300**_**-DVD**	85.8 ± 30.1	0.8 ± 0.02	68.2	6.11
**PLLA (pristine)**	–	–	5.6	0.5

**Fig. 6:**
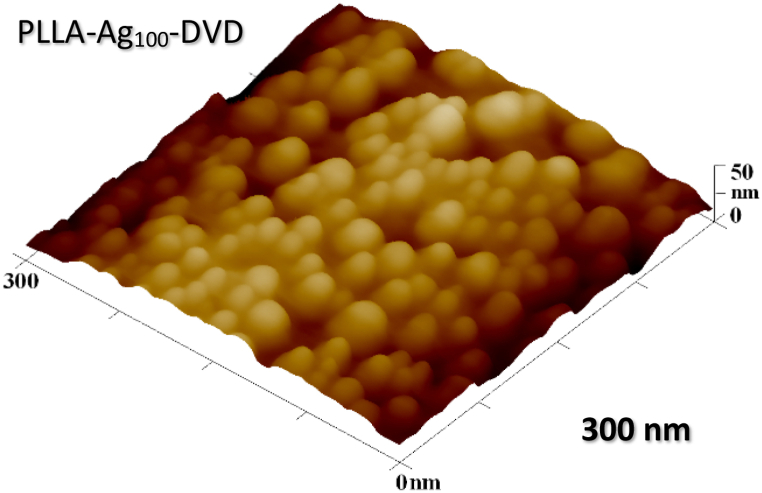
Detailed AFM surface image of silver clusters on top of ridge of 100 s deposited PLLA based sample.

As it is obvious from detailed AFM image (Fig. 6), granular nanostructures appeared on the Ag_100_ deposited surface – non-continuous silver layer consolidated itself into small silver clusters, increasing the overall surface rougness and effective surface area and creating dual scaled, multi-hierarchial structures.

**Fig. 7:**
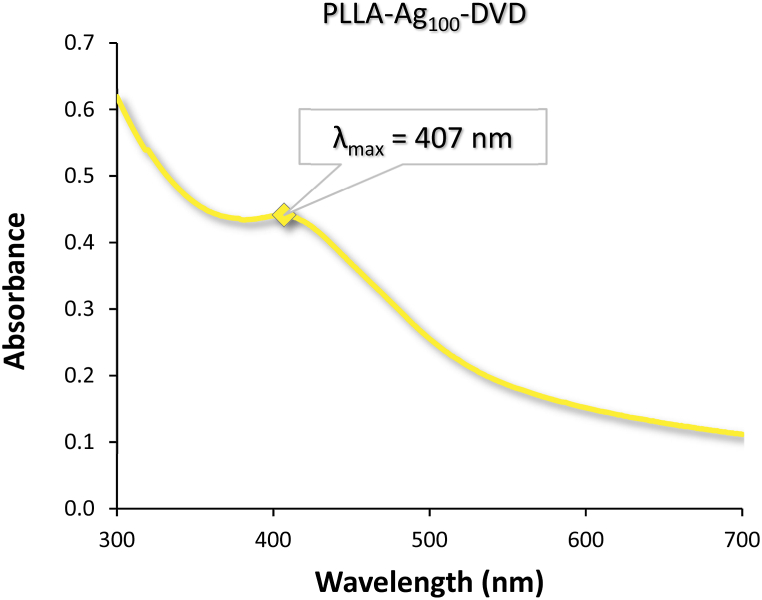
A simple UV–Vis absorption spectrum displaying the peak absorption wavelength (*λ*_max_) for silver nanoparticles embedded on surface of PLLA-Ag100-DVD sample.

It can be concluded, there seems to be thickness (or rather continuity) threshold above which the silver nanoparticles don't appear upon annealing. Thin silver films of higher thicknesses remain whole and keep their non-transparent silver reflective colour of a bulk material as they are embedded onto PLLA surface. Films of the lower thicknesses, on the other hand, congregate into small silver globular clusters upon annealing and are embedded into PLLA as such, changing the surface colour to yellow yet remaining transparent. Presence of silver nanoparticles was verified by UV–Vis spectrophotometry with the typical absorbance peak for silver nanoparticles being approximately around wavelength of 380–410 nm for colloidal solutions [42], manifested by yellow colour, is presented by Fig. 7 with maximum at 407 nm. The ability to converge into clusters belongs to non-continuous films, and stems from the stages of conventional thin film growth (nucleation – island growth – coalescence – continuous film formation) [43]. Partial immersion of the silver nanoparticles in the polymer matrix makes the PLLA-Ag_100_-DVD sample a hybrid metal-polymer nanocomposite, a recently emerging and innovative class of materials [44].

The silver layer, however, considerably decreases the precision of the replication and lowers the height of the pattern. Thermal annealing of thicker continuous metal films on polymer film also creates thermal expansion strain on the bilayer interface, which can result in secondary wrinkling upon cooling, as can be observed on PLLA-Ag_300_-DVD sample, evident on Fig. 5.

### 3.3 Wettability

**Fig. 8:**
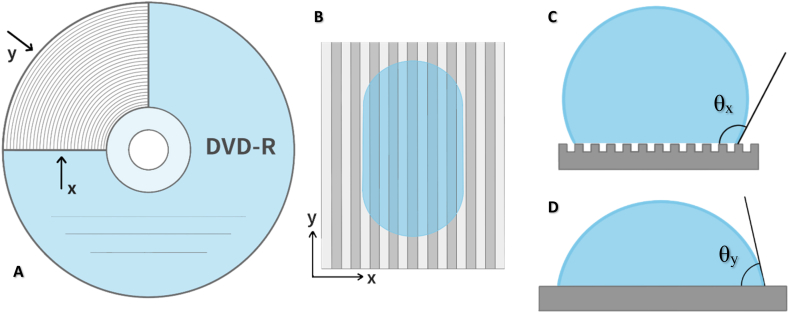
Illustration of the concentric grooved pattern on optical discs and the direction of wettability measurement (A). A schematic top view of water droplet distortion (B) and the contact angle profile of θx (perpendicular) (C) and θy (parallel) (D).

The surface topography also affects its wettability, which may be contributing to antibacterial performance, but is not necessary in correlation. Anisotropic wetting suggests inhomogeneous dispersal of liquid droplet in different directions, usually caused by presence of ordered periodic surface structures with heterogeneity in one dimension (e.g. parallel lines, grooves, wrinkles). This phenomenon includes both static properties (different static CAs in different directions, Fig. 8) and dynamic properties (different sliding angles in different directions). As presented below, the difference in CAs measured parallel to the groove direction (θ_y_) and CAs measured perpendicularly to the grooves (θ_x_) was observed for both PDMS and PLLA nanostructured surfaces. The degree of wetting anisotropy can be defined as Δθ = θ_x_- θ_y_ [7], and is, as the droplet distortion, affected by the volume of the measurement droplet.

**Fig. 9:**
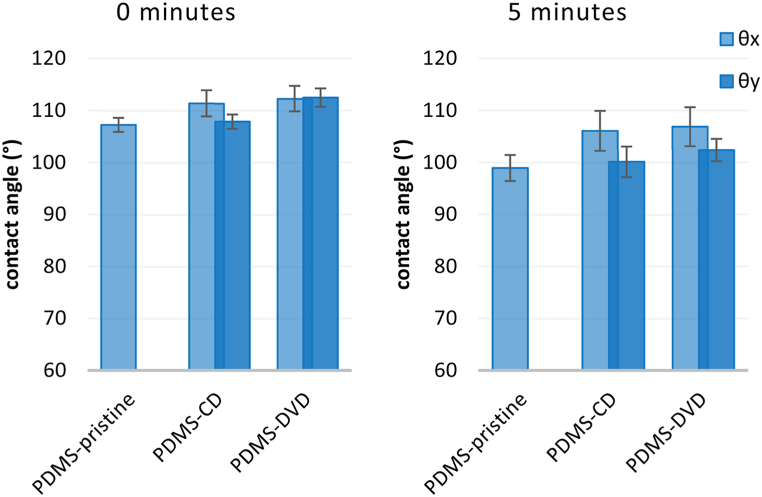
Wettability of PDMS based patterned surfaces (compared with non-patterned) measured immediately after drop landing and after 5 min.

**Fig. 10:**
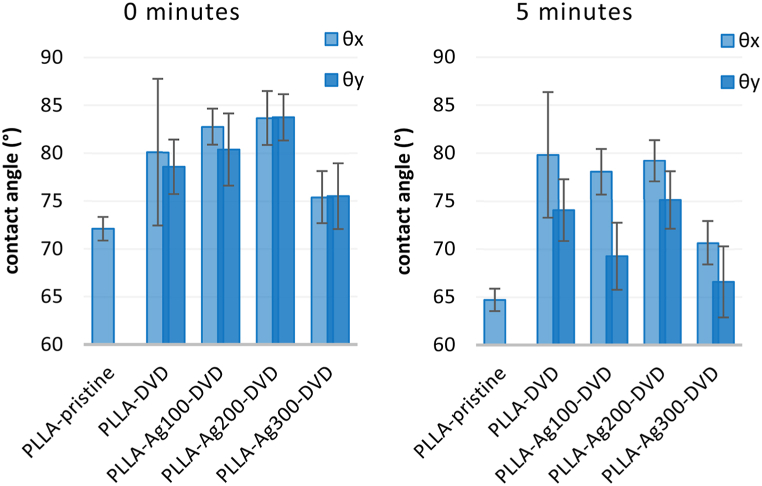
Wettability of PLLA and PLLA-Ag based patterned surfaces (compared with non-patterned) measured immediately after drop landing and after 5 min.

**Fig. 11:**
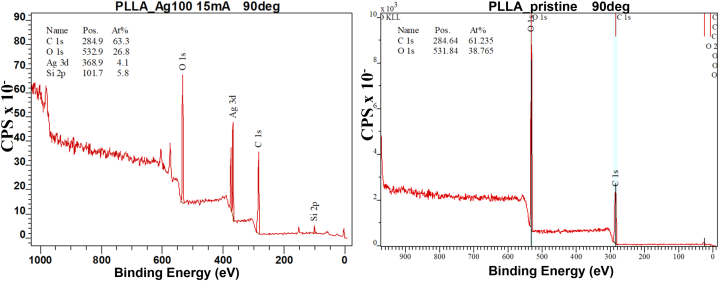
XPS spectrum of a silver enhanced and nanostructured PLLA foil (left) compared to spectrum of original pristine PLLA foil (right), measured under 90° angle.

The increase of anisotropic effect in time is driven by capillary forces and in part by surface tension of the measuring liquid. The anisotropic effect was less predominant on PDMS surfaces (Fig. 9), due to high natural hydrophobicity of PDMS, but was more prominent on PLLA surfaces, especially without silver. Silver deposition slightly changed surface chemistry, but primarily lowered the precision of submicron structure replication from PDMS mold. The increase in silver film thickness decreases the height of resulting nanostructures and the shallower grooves therefore evince lower capillary thus anisotropic behavior of the surfaces. With the rise of the surface elemental silver concentration, the eventual wettability also slightly increases (Fig. 10) (see Fig. 11).

Nonetheless, we can determine, that the overall wettability of surfaces decreased with the nanostructuring and the increase of surface roughness, which is well-known and well-explored phenomena.

The antibacterial activity of textured surfaces is hinged on degree of bacterial adhesion, which encompasses an initial bacterial attraction and subsequent attachment. There are many factors, that can influence the bacteria attachment to a given substrate, namely cell surface hydrophobicity and surface charge. Based on the bacteria surface and cell wall composition, the physical forces can either increase or decrease the proclivity to adhesion. Bacteria are predominantly hydrophilic, therefore are prone to adhere to hydrophilic surfaces more easily. Cell surface charge can also influence the scope of bacterial attachment in between species, although most bacterial strains exhibit negative cell charge by virtue of an excess of carboxyl and phosphate groups in their cell walls. The positively charged nanostructured surfaces hence possesses greater antibacterial activity and selectivity, as a result of the stronger interactions with negatively charged bacterial membranes [45]. In our case, the readily formed positively charged Ag^+^ ions diffuse into medium, leaving the bulk at net negative charge at the surface, but creating positively charged subsurface region and immediate area above surface with high concentration of silver ions.

### 3.4 Surface chemistry of the PLLA replicas

The changes in chemical composition of nanostructured and silver enhanced samples were studied by X-ray photoelectron spectroscopy (XPS) and the results are summarized in Table 3 below. The XPS analysis shows, that by thermal annealing and resulting nanostructuring, the chemical composition of sample surface does not significantly change.

**Table 3:** Atomic concentration of the sample surface obtained by the XPS method, the analysis was performed by detecting the outgoing beam at angle of 14° and 90° (perpendicular to the sample surface). Antibactericidal efficacy was evaluated by comparison to the control group after 4 h, for *Escherichia coli* (EC) and *Staphylococcus epidermidis* (SE).

Sample	Carbon concentration (%)		Oxygen concentration (%)		Silicon concentration (%)		Silver concentration (%)		Antibacterial efficacy after 4 h (%)	
	90°	14°	90°	14°	90°	14°	90°	14°	EC	SE
**PLLA – DVD**	58.7	58.1	37.6	30.6	3.6	11.3	–	–	−7.8	48.1
**PLLA-Ag**_**100**_**-DVD**	63.3	57.0	26.8	22.8	5.8	15.7	4.1	4.6	81.5	79.1
**PLLA-Ag**_**200**_**-DVD**	52.3	56.5	14.6	13.7	3.2	7.3	24.1	17.2	−1.8	70.7
**PLLA-Ag**_**300**_**-DVD**	54.8	54.8	13.0	14.8	2.1	8.1	24.1	12.0	12.3	78.6
**PLLA (pristine)**	61.2	65.9	38.8	34.1	–	–	–	–	17.0	48.1

However, when PLLA surfaces were annealed on silver deposited PDMS molds, the final nanostructured PLLA films exhibit decrease of oxygen levels on top of the presence of silver and residual silicon from PDMS mold. The reduction of oxygen levels is probably the result of Ag_2_O formation and the surface carbonyl group content loss. The peaks on the left side of O1s peak represents Ag 3p, at the positions 574.5 eV and 604.9 eV.

### 3.5 Antibacterial performance of the patterned films

**Fig. 12:**
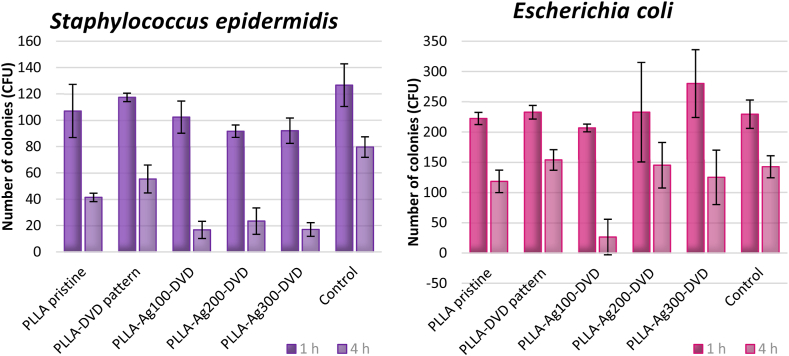
Antibacterial activity of PLLA samples against bacterial strains of *S. epidermidis* and *E. coli* after 1 and 4 h incubation. Samples: pristine PLLA foil, DVD patterned PLLA, DVD patterned PLLA enhanced by silver (deposition times of 100, 200 and 300 s, at 15 mA). CFU represents colony forming units grown on agar plates.

**Fig. 13:**
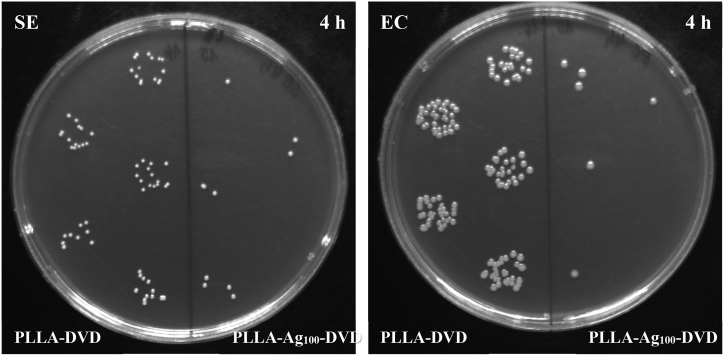
Representative photos of CFUs for DVD patterned PLLA without silver (left half of Petri dish) and with silver nanoparticles (right side of Petri dish), for both bacterial strains after 4 h incubation.

Next, we also determined the antibacterial properties of both nanopatterned PLLA and Ag-reinforced nanopatterned PLLA and compared them to properties of the flat pristine PLLA foil. The antibacterial activity was evaluated against two bacterial strains: Gram-positive *S. epidermidis* and Gram-negative *E. coli*. As it is obvious from Fig. 12, the most prevalent antibacterial effects were exhibited by Ag-doped patterned samples mostly against *S. epidermidis*, whereas the highest bactericidal effect was achieved by the PLLA-Ag_100_-DVD nanopatterned nanocomposite for both bacterial strains (for representative image see Fig. 13). That corresponds with the increase in effective surface area and also with nanoparticulation of silver, which accelerates the silver dissolution and subsequent Ag^+^ ion release, promoting the antibacterial effect. However, contrary to the hypothesis, the nanopatterned PLLA-DVD foil demonstrated slightly increased bacterial growth in comparison to the non-patterned PLLA foil, which may be caused by the increased surface roughness and therefore more feasible bacterial attachment or wedging between structures. After 24 h incubation, all silver doped samples were proven to be completely bactericidal.

It can be concluded, that the presence of silver grants the surface biocidal properties and the performance is closely connected to the quantity of silver accessible on the surface and also its form.

**Fig. 14:**
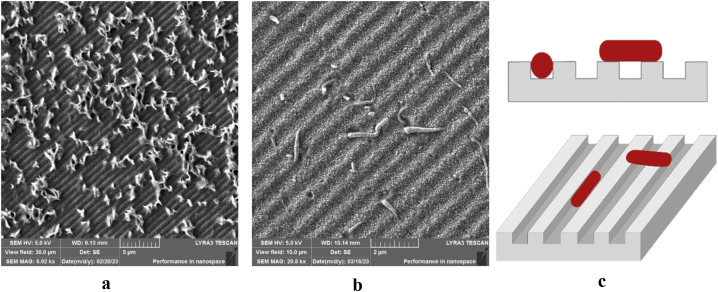
(a)SEM image of *S. epidermidis* attachment on DVD patterned PLLA surface. (30 μm) Fig. 14b. SEM image of *E. coli* settlement on surface of PLLA-Ag200-DVD sample. (10 μm) Fig. 14c. Illustration of bacterial (*E. coli*) settlement between and on top of surface structures.

The dimensions and shape of nanostructures are another critical parameter in assessing bactericidal activity. Height and spacing between surface features, notably combined with high aspect ratio, influence the antibacterial activity (see Fig. 14 a-c). In particular, when structures possess spacing larger than bacterial diameter, the bacteria tend to settle between the structures, to maximize the contact area [46], instead of interacting with tips of the pattern, which can therefore lead to increased proliferation [47]. According to available data in literature, the dimensions of rod-shaped *E. coli* are 1–3 μm in length and 0.4–0.7 μm in diameter [48], whereas the spherically shaped *S. epidermidis* has diameter of 0.5–1 μm [49]. Herein fabricated and described topographical structures can therefore demonstrate favourable conditions for bacterial settlement in grooves for smaller and adequately spatially rotated bacteria. That can be, however, detrimental in case of silver enhanced surfaces, considering the bacteria's closer contact with surface and the higher concentration of active silver ions in surface proximity. Herein presented SEM images (Fig. 14a and b) show bacterial settlement and the change in bacterial morphology after rupture or lysis and subsequent drying, resulting in loss of mass and typical healthy state shape [50,51], on bacteria shape and morphology also silver with piezoelectric substrate may have a strong influence [52]. Also subsequent effects, such as presence of heterojunction may play an important role [53].

Periodic nanogrooves of these dimensions are, nonetheless, also favourable for cell attachment, growth, differentiation and alignment as reported by Wu et al. [54] on muscle cells, therefore convenient for surface development for tissue engineering, also with possibility of cell nerves enhancement [55]. Based on the experimental design, the anisotropic effect might also play a role in antibacterial observations in dynamic or static medium. Generally, anisotropic nanostructures are reported to be more effective against gram-negative bacteria strains, although favourable activity is reported also against gram-positive bacteria [56].

## 4 Conclusions

We have successfully prepared replicated biopolymer pattern from poly-l-lactic acid doped with silver nanoparticles. The replication process consisted from two basic steps. The first procedure was based on the replication of the pattern from commercially available CD and DVD discs, where the successful replication into PDMS was verified by morphology determination. The replicated pattern into PDMS was subsequently used for biopolymer replication, where the process was firstly verified by simple replication of the PDMS into pristine PLLA foils. The replica, their dimensions, surface morphology and chemistry we again verified with the high similarity both to PDMS master and also the pattern original version. Finally, the PLLA foils were covered with thin Ag nanolayers and the replication process was then repeated in the same manner as for pristine PLLA. During the replication process the silver formed isolated nanoclusters, which was visually confirmed by the change of the foil into yellow colour, and by LSPR peak in UV-VIS spectrum, which was detected on the position typical for Ag nanoclusters. The PLLA replicated patterns with silver nanoparticles exhibited strong antibacterial properties for both G+ and G-bacteria. The inclusion of silver nanoparticles and thin silver films aim at slow releasing silver ions close to the surface. These ions then can reduce or even prevent bacterial colonization and subsequent biofilm formation on the surface.

## CRediT authorship contribution statement

**Bára Frýdlová:** Investigation, Writing – original draft, Writing – review & editing. **Dominik Fajstavr:** Data curation, Formal analysis, Investigation. **Nikola Slepičková Kasálková:** Conceptualization, Investigation, Writing – original draft, Methodology. **Silvie Rimpelová:** Formal analysis, Investigation, Validation, Methodology. **Vladimíra Svobodová Pavlíčková:** Data curation, Investigation. **Václav Švorčík:** Data curation, Formal analysis, Resources. **Petr Slepička:** Conceptualization, Data curation, Funding acquisition, Investigation, Methodology, Supervision, Writing – original draft, Writing – review & editing.

## Declaration of competing interest

The authors declare that they have no known competing financial interests or personal relationships that could have appeared to influence the work reported in this paper.
